# Automated procedure to detect subtle motor alterations in the balance beam test in a mouse model of early Parkinson’s disease

**DOI:** 10.1038/s41598-024-51225-1

**Published:** 2024-01-09

**Authors:** Raphaëlle Bidgood, Maider Zubelzu, Jose Angel Ruiz-Ortega, Teresa Morera-Herreras

**Affiliations:** 1 Department of Pharmacology, Faculty of Medicine and Nursing, University of the Basque Country (UPV/EHU), Barrio Sarriena S/N, 48940 Leioa, Biscay Spain; 2 Autonomic and Movement Disorders Unit, Neurodegenerative Diseases, Biobizkaia, Barakaldo, Biscay Spain

**Keywords:** Parkinson's disease, Neurodegeneration

## Abstract

Parkinson’s disease (PD) is the most common motor neurodegenerative disorder, characterised by aggregated α-synuclein (α-syn) constituting Lewy bodies. We aimed to investigate temporal changes in motor impairments in a PD mouse model induced by overexpression of α-syn with the conventional manual analysis of the balance beam test and a novel approach using machine learning algorithms to automate behavioural analysis. We combined automated animal tracking using markerless pose estimation in DeepLabCut, with automated behavioural classification in Simple Behavior Analysis. Our automated procedure was able to detect subtle motor deficits in mouse performances in the balance beam test that the manual analysis approach could not assess. The automated model revealed time-course significant differences for the “walking” behaviour in the mean interval between each behavioural bout, the median event bout duration and the classifier probability of occurrence in male PD mice, even though no statistically significant loss of tyrosine hydroxylase in the nigrostriatal system was found in either sex. These findings are valuable for early detection of motor impairment in early PD animal models. We provide a user-friendly, step-by-step guide for automated assessment of mouse performances in the balance beam test, which aims to be replicable without any significant computational and programming knowledge.

## Introduction

Parkinson’s disease (PD), the most common motor neurodegenerative disorder, is characterised by the progressive loss of dopaminergic (DA) neurons in the *substantia nigra pars compacta* (SNc) and the presence of intracytoplasmic inclusions of α-synuclein (α-syn), called Lewy bodies^1–6^. It is an incurable disease with a diagnosis based on clinical criteria and the appearance of motor symptoms^5^. A handicap in PD therapeutics is that the cardinal motor symptoms (bradykinesia, resting tremor, muscular rigidity and postural instability) appear when approximately 40–60% of DA neurons have died and 30–40% of striatal DA content has been lost^7^. Consequently, at that point, any possibility of delaying disease progression or achieving neuroprotection may already be out of reach.

The development of new disease-modifying therapies for PD is critically dependent on animal models. In this line, a significant loss of DA often needs to occur in animal models of PD in order to observe changes in motor function^8^. Identifying reliable signs of motor impairment as early as possible would be of great importance to be able to allow the preclinical evaluation of potential therapeutic agents^9^. Thus, a wide range of behavioural tests have been developed to investigate motor impairments and, among them, the balance beam test is particularly useful for detecting subtle deficits in motor skills and balance that may not be detected by other motor tests^10^. This task is effective in measuring fine coordination as well as balance, and can be used for early detection of motor impairment in PD mouse models^11^.

Animal performances in the balance beam test can be quantified manually by measuring the time it takes for the mouse to traverse the beam, the maximum distance covered, the number of times a hind paw comes off the top of the beam, and the number of falls^10,11^. Nevertheless, manual analysis of rodent behaviour is limited by the reproducibility and repeatability of the findings due to low levels of inter-rater reliability between different observers^12^, as well as being highly labour intensive, susceptible to observer drift and limited to our senses^13,14^. Recent advances in the field of computational neuroethology have made it possible to automate animal behaviour analyses by capturing animal postures (pose estimation) and predicting behavioural patterns (behavioural classification) in recorded videos^13,15,16^. Such powerful tools are transforming research in terms of animal tracking, behaviour detection and classification, by utilising advances in computer vision, deep learning and computational algorithms like deep neural networks^12,17^. However, using these automated methods often require extensive computational knowledge and programming skills^14^.

DeepLabCut is an open-source toolbox based on transfer learning that enables to train a deep neural network with a small amount of annotated data to track user-defined features to monitor animal behaviour, and reach human labelling accuracy^18^. DeepLabCut toolbox provides a graphical user interface (GUI) aiding researchers with minimal programming skills to label body-parts, train, evaluate the network, and analyse novel videos for pose estimation. Animal tracking output files of DeepLabCut can then be loaded into other programs, such as Simple Behavior Analysis (SimBA), for further analysis. SimBA is an open-source method with a GUI that generates random forests algorithms to accurately classify behavioural patterns, using poses extracted from videos by DeepLabCut and user-made annotations. SimBA was developed to create supervised machine learning predictive classifiers of rodent social behaviour, but is adaptable to study an extensive set of behaviours in various experimental protocols^14^.

Here, we aimed to combine the conventional manual analysis approach of mouse performances in the balance beam test with computational neuroethology methods to create automated models that can track mouse body-parts in recorded videos and predict the presence of walking along the beam as well as the pattern of that behaviour. We compared the manual scoring with our automated procedure for the time to cross the beam, the maximum distance covered and the number of falls. We also provide a user-friendly, step-by-step guide with a replicable and flexible protocol to track walking behaviour in the balance beam test in a mouse model of PD.

## Material and methods

### Animals and ethics statement

A total of 47 wild type male (n = 23) and female (n = 24) C57BL/6J mice (Janvier Labs) were used. Mice were 7 weeks old at the beginning of the experiment and were housed in groups of 4 in individually ventilated cages under standard laboratory conditions (22 ± 1 °C, 55 ± 5% relative humidity, and 12:12 h light/dark cycle) with food and water provided ad libitum. Animal studies were reported in compliance with the ARRIVE guidelines. The experimental protocol was reviewed and approved by the Local Ethical Committee for Animal Research of the University of the Basque Country (UPV/EHU, CEEA, ref. M20-2022-215). All of the experiments were performed in accordance with the European Community Council Directive on “The Protection of Animals Used for Scientific Purposes” (2010/63/EU) and with the Spanish Law (RD 53/2013) for the care and use of laboratory animals.

### Adeno-associated viral (AAV) vectors and surgical procedure

**Figure 1:**
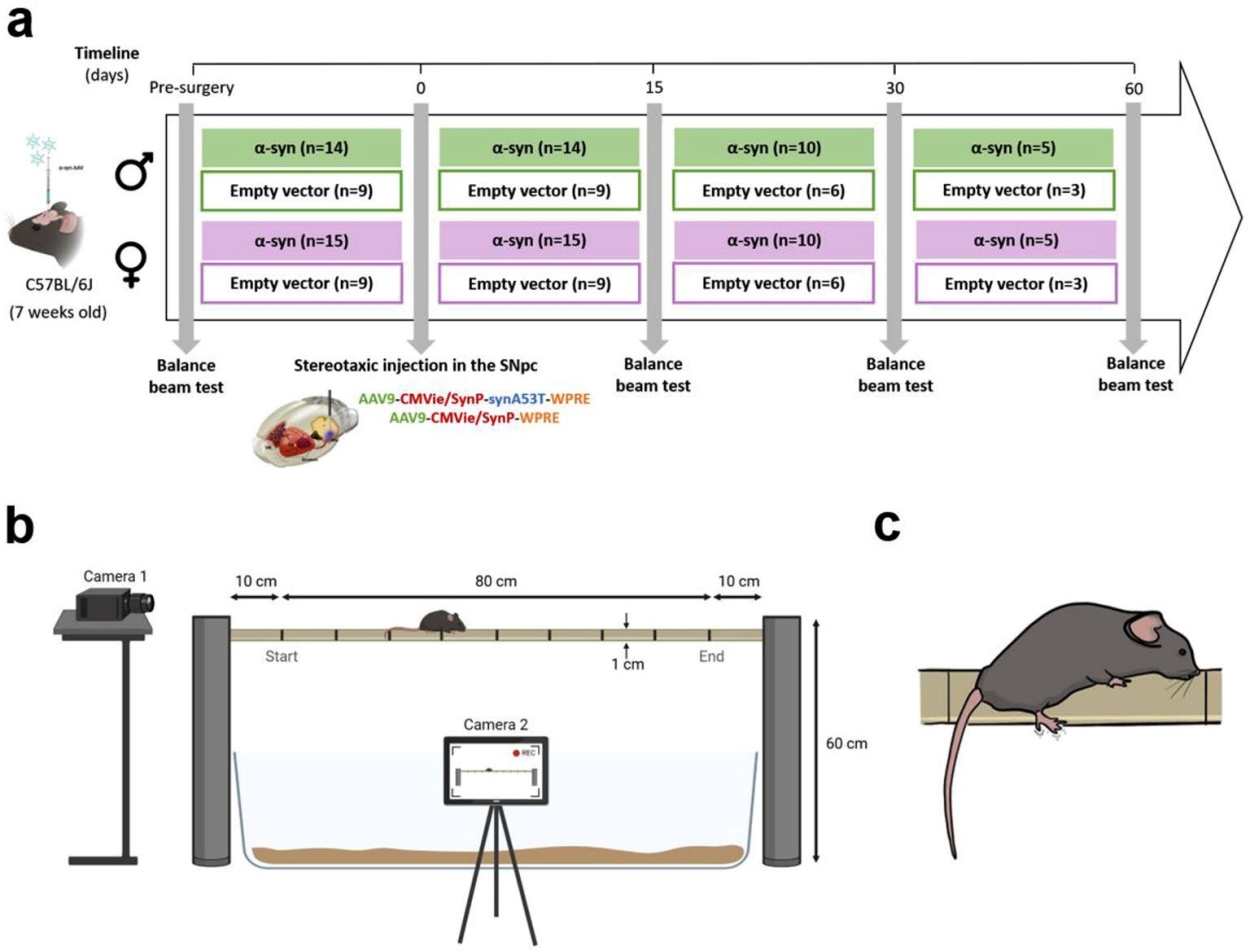
Experimental layout. (**a**) Description of the experimental groups and time points of the behavioural test. Animals were bilaterally injected with AAV9-A53T-h-α-syn or with the empty vector in the SNc (male and female α-syn or empty vector groups, respectively). (**b**) Schematic representation of the experimental setup of the balance beam test, assessing the capability of the mouse to walk across the wooden beam (80 cm of length and 1 cm of width at a height of 60 cm). Cameras 1 and 2 (placed at the rear and laterally to the beam, respectively) recorded each trial for in-depth manual and automated analyses, post-behavioural sessions. (**c**) Illustration of a hind paw slip, defined as the back foot coming off the top of the beam.

Adeno-associated viral vectors (AAV9-CMVie-SynP-WPRE) inducing the overexpression of either A53T mutated hα-syn (1 × 10^13^ genomic particles/ml) or the empty vector (3.1 × 10^12^ genomic particles/ml) were obtained from the University of Bordeaux (France). Mice were anaesthetised with isoflurane in oxygen-enriched air (1–2%) and placed in a stereotaxic frame (David Kopf^®^ Instruments). The corresponding AAV vectors were bilaterally injected into the SNc (1 µl per hemisphere) with a glass pipette [coordinates from Bregma: anteroposterior (AP) − 2.9 mm; mediolateral (ML) ± 1.4 mm and dorsoventral (DV) − 4.5 mm]. The pipette was left in place for 5 min after the injection to avoid leakage. Motor performances in the balance beam test were evaluated at pre-surgery and at 15, 30 and 60 days post-surgery. The design and timeline of the experiments is shown in Fig. 1a.

### Evaluation of motor performance: balance beam test

The beam walking assay in rodents offers the possibility to detect subtle deficits in motor skills and balance that other motor tests such as the Rotarod, may not be able to detect. The task assesses the capability of an animal to walk across a wooden beam of 80 cm of length and 1 cm of width at a height of 60 cm (Fig. 1b). The mouse is placed before the start line and is removed from the beam once it has crossed the finishing line or when the time on the beam has exceeded 1 min. Lines were hand-marked every 10 cm on the beam to measure the maximum distance covered by the mouse, which was achieved by counting the number of lines crossed during a trial. Animals were given 3 trials, each separated by 10 min. Before the first trial, mice were acclimated to the behavioural room for 30 min and 1 day prior to testing, mice were trained to cross the beam in the same conditions as the testing (3 trials per animal, with a 10 min interval between each trial). Only the testing sessions were recorded for in-depth analyses combining manual and automated methods.

Each trial was recorded by two cameras for manual and automated analyses (cameras 1 and 2, Fig. 1b). Camera 1 was placed at the rear of the beam to show a back view of the mouse crossing the beam for quantification of hind paw slips. A slip is defined as the foot coming off the top of the beam (Fig. 1c). Camera 2 was placed laterally to the beam to record a side view of the whole length of the beam, to manually measure the time before the start line (i.e. the time before the animal starts the test by crossing the start line, once it has been placed onto the beam), the time to cross the beam, the distance travelled and the number of falls, and for automated analyses using DeepLabCut and SimBA.

### Video recordings and pre-processing

Videos were recorded with 30 frames per second (fps) with frame dimensions of 1280 × 720 (resolution width and height, respectively). To reduce computational load, videos were pre-processed before being used in DeepLabCut. All video files surpassing 1 min were trimmed to not exceed that time limit, as set by the chosen default parameters of the balance beam test. Videos were also trimmed to only show the trial, thus excluding the placement of the animal onto the beam by the experimenter and the time up until the animal crosses the start line.

### Pose estimation using DeepLabCut

#### Installation

DeepLabCut (version 2.2.3.) was installed in a conda environment, according to the detailed steps described on the GitHub page (https://github.com/DeepLabCut/DeepLabCut/blob/main/docs/installation.md) and was run on a Windows 11 operating system, with Anaconda Navigator (anaconda3, version 2.4.0) and TensorFlow (version 2.11.0) installed. Visual Studio Code (version 1.77.3) was also set up to modify .yaml files.

#### Project creation and frame extraction

A single animal project was created in the DeepLabCut Graphical User Interface (GUI) with 16 videos from different behavioural sessions, providing representative examples of diverse set of behaviours, animal size, room lighting and camera distance variabilities to increase neural network training efficiency. The corresponding configuration (config.yaml) file was modified to include the names of the following 5 body-parts: “Nose”, “Head”, “Bodytop”, “Bodymiddle”, “Tailbase” (operational definitions of mouse body-parts are shown in Supplementary Table 1). Default parameters were used to extract 320 frames (20 frames per video), using the automatic method in DeepLabCut. The k-means algorithm was selected to cluster the frames based on visual appearance, due to the sparse nature of the mouse walking behaviour across the beam in certain videos. This function downsamples videos, clusters the frames and selects frames from different clusters, to generate different-looking frames.

#### Labelling frames

All extracted frames were next used to manually annotate 5 body-parts on the mouse in the labelling toolbox in the interactive DeepLabCut GUI. Invisible or occluded body-parts in a frame were not labelled by skipping to the next body-part, in order to generate a model that only applies a label to a visible body-part. The check_labels function was then used to verify the labelling accuracy in the annotated frames (position of the keypoints).

#### Training and evaluating the network

The open-access Google Colaboratory Notebook for standard single-animal projects was used to train the network (DeepLabCut version 2.3.2, Python version 3.9.16), while the evaluation step was run locally, on the DeepLabCut GUI. Prior to training, the labelled datasets from the 16 videos were combined and then split into “train” and “test” datasets using the create_training_dataset function (resnet-50 pre-trained network weights and imgaug augmentation method). The training fraction was set to 0.95, so that the neural network was trained with 95% of the 320 extracted frames and the performance of the trained network was evaluated with the remaining 5% (all body-parts were compared). During training, loss values were displayed in the terminal at every 1000th iteration and the (intermediate) weights of the network (checkpoints) were stored every 5000 iterations, using the save_iters function.

#### Novel video analysis and creation of labelled videos

Videos unknown to the DeepLabCut model were analysed with the default parameters to use the most recent checkpoint (last). Output data were saved in a CSV file format for further computational analyses. A skeleton interconnecting the keypoints was built and included in the created labelled video to visually double check labelling accuracy (Supplementary Fig. 1a). Pose estimation results were plotted in the DeepLabCut GUI for each video analysed by the model (example shown in Supplementary Fig. 1b–d). The desired level of performance of the model was reached without optional refinement.

### Walking classifier prediction using Simple Behavior Analysis (SimBA)

#### Installation

SimBAxTF-development (version 1.61.3) was installed in a conda environment with Python 3.6 and FFmpeg, according to the documentation on GitHub (https://github.com/sgoldenlab/simba/blob/master/docs/anaconda_installation.md).

#### Project creation

A new project was created in the main SimBA console, following Scenario 1 (https://github.com/sgoldenlab/simba/blob/master/docs/Scenario1.md). Prior to generating the project config file, a new user-defined pose config file was created, to match the labels in the DeepLabCut config.yaml file (tracking 1 animal and 5 body-parts: “Nose”, “Head”, “Bodytop”, “Bodymiddle”, “Tailbase”). To define the body-parts, an image was selected from the extracted frames of the DeepLabCut project folder, to place all 5 keypoints on the animal. Once body-part locations were assigned, the user-defined pose config file was selected in the animal settings menu. The project config file was generated with 1 predictive classifier (behaviour), named “walking”, and with the default classical tracking in the type of tracking menu (as mice are clearly distinguishable by eye in the videos). A detailed operational definition for the classified behaviour is provided in Supplementary Table 2, with behaviour description and characteristic initiation, duration and end.

#### Importing videos and tracking data

16 videos (in mp4 format) and the associated tracking data files (DeepLabCut CSV output files) were imported into the SimBA project folder. These videos corresponded to the initial 16 representative videos selected for creating the DeepLabCut model, and were used for behavioural annotation, classification and analysis in SimBA. An additional video with the associated tracking data was later imported into the SimBA project for the optional validation step, as recommended by the SimBA documentation on GitHub (https://github.com/sgoldenlab/simba/blob/master/docs/tutorial.md#optional-step-before-running-machine-model-on-new-data).

#### Loading the project

The project config was loaded by selecting the *project_config.ini* file in the project folder and for each video, meta parameters were specified (frame rate (fps), resolution and metric distances) and pixel measurements were set by calculating the pixels per millimetre from a known (real life) distance between two points. The outlier correction step was skipped and features used for behavioural classification were extracted in SimBA. Walking behaviour was manually labelled for the 16 videos (i.e. annotated as “present” when visible in a frame), before training a single machine model for behavioural classification. The SimBA model was built using a random forest machine model with 2000 estimators, and trained on 80% of our data (test size 20%) (see Supplementary Fig. 2). As random forest classifiers are sensitive to class imbalances, the random undersample setting was selected with an under-sample ratio of 1, to prevent class imbalances that occur when observations of the majority class (absence of the classified behaviour) outweigh observations of the minority class (presence of walking behaviour). Custom class weights were set to assign twice the importance of behaviour present annotations (“walking” present) over behaviour absent annotations (“walking” absent). Model evaluation settings were set to create model meta data files (list of hyper-parameter settings used when creating the model), a classification report, precision recall curves and a feature importance bar graph with 15 features (Supplementary Figs. 2, 3).

#### Validation of the model on single video

The SimBA model was validated by determining the optimal discrimination threshold as well as the minimum length of a classified behavioural bout (corresponding to the minimum duration of uninterrupted walking) on a video that was not used to train the model. This “gold-standard” video was fully manually annotated for the behaviour of interest but was not included in the training dataset. A validation video was created with a gantt chart to verify the correct detection of the behavioural bouts by the model.

#### Running the machine model

Prior to running the SimBA model to do machine predictions, the model was set with the following settings: classifier: walking; model path: walking.sav path; discrimination threshold: 0.8; minimum bout length (ms): 100. Machine predictions were then analysed in SimBA to generate descriptive statistics of the predictive classifier in the project. Gantt plots and classification probability plots were generated in the Sklearn visualisation tab for all the videos in the project.

### Falls classifier prediction using SimBA

Following the same pipeline previously described for creating an automated model in SimBA to detect the presence of walking, a new project was created for predicting falls (operational definition for the falls classifier is provided in Supplementary Table 2). The model was built using 11 videos, with falls manually labelled as being present or absent in each video frame. Videos were selected to reflect different types of falls varying in their duration (e.g. pronounced slips with both hind paws below the beam, or the animal being upside down still clinging onto the beam). The model was trained with the same settings previously described (random forest algorithm with 2000 estimators; test size of 0.2; random undersample with an undersample ratio of 1; custom class weights set to 2 for falls present and 1 to falls absent) and was evaluated to assess its performance (Supplementary Fig. 4). A validation video was used to determine the optimal discrimination threshold (set to 0.92) and the minimum bout length (corresponding to the minimum duration of fall, set to 1400 ms), which were then used to analyse all the videos and generate machine predictions.

### Regions of interest (ROIs) in SimBA

The SimBA ROI interface was used to draw 10 ROIs along the beam, corresponding to the regions before the start line and after the finishing line, and the 8 other ROIs were each delimited by the hand-drawn lines along the beam (marked every 10 cm) (illustrated in Supplementary Fig. 5). The rectangular shapes were drawn once and applied to all the videos analysed in SimBA. Some ROIs were slightly adjusted to correspond to the sectioned areas of the beam previously defined, if some changes in the camera angles were detected. SimBA provided the number of entries in each ROI as well as the time spent in each of them, which was used to determine the time to cross the beam (s) and the maximum distance covered (cm) for comparison with the manual approach. Animals entering a ROI at least once were considered to have crossed the corresponding line, aided to calculate the maximum distance covered (i.e. the distance associated with the furthest line from the start line that was crossed by the animal). Values of the times spent in each ROI of the beam were summed to determine the time to cross the beam. Quantifying the time before the start line (s) was not included in our automated procedure as videos had been pre-processed to only show the entirety of the trial (beginning when the animal crosses the start line). The ROI data were analysed in SimBA using the body-part “Bodymiddle” as a reference, corresponding to the closest keypoint to the hind paws, used in the manual analysis to decide whether an animal crossed a line or not.

### Data exclusions

Videos of mice never crossing the start line (< 12%) were excluded from the DeepLabCut and consequently, the SimBA analyses as these subjects never walked along the beam in the balance beam test. However, these videos were used for the manual quantification analysis (time before the start line, time to cross the beam, maximum distance travelled), excluding the slip counts and the manual quantification of falls. As for the ROIs in SimBA, videos excluded from the DeepLabCut analysis were assigned the default values of a failed trial (a time to cross the beam of 60 s and a maximum distance covered of 0 cm) as these animals never crossed the start line.

### Evaluation of α-synuclein overexpression and nigrostriatal degeneration

#### Tissue processing

Animals were deeply anaesthetised with 0.2 ml xylazine and 0.6 ml ketamine i.p. and transcardially perfused with saline through the ascending aorta, followed by 4% paraformaldehyde in 0.1 M phosphate buffered saline (PBS). Fixed brains were coronally sliced at 40 μm by a freezing microtome (HM 430, Microm), and stored in a cryoprotective solution at − 20 °C until further processing.

#### Immunohistochemistry

α-Syn and tyrosine hydroxylase (TH)-immunostainings were used to corroborate the correct overexpression of hα-syn using AAV vectors and to examine the degree of nigrostriatal DA denervation, respectively. These immunostainings were performed on free-floating striatal and nigral sections using antibodies raised against α-syn (mouse, 1:10,000, Abcam, ref. AB27766) and TH (rabbit, 1:1000, Merck, ref. AB152). Sections were respectively rinsed three times in PBS or potassium-phosphate buffer (KPBS) between every incubation period. All incubation solutions contained 0.5% Triton X-100 in PBS or KPBS. The sections were quenched for 30 min in a mixture of 3% H_2_O_2_ and 10% methanol and pre-incubated for 1 h with 5% normal horse serum (NHS). Thereafter, sections were incubated overnight at room temperature (RT) with the corresponding primary antibody in 5% NHS. Sections were then incubated for 2 h at RT with a biotinylated secondary antibody (universal, 1:200, Vector Laboratories, ref. PK6200; horse anti-rabbit, 1:200, Vector Laboratories, ref. BA1100-1.5) in 2.5% NHS, followed by avidin–biotin–peroxidase complex (ABC Elite, 1:200, Vector Laboratories, ref. PK-6100). Peroxidase activity was visualised using 3,3′-diaminobenzidine and H_2_O_2_, and finally, sections were mounted, cleared with xylene and coverslipped using DPX mounting medium.

#### Optical densitometry analysis

Striatal and nigral sections were optically digitised using an EPSON V700 scanner at a resolution of 6,400 ppp. Densitometry analyses of TH + fibres in the striatum and *substantia nigra pars reticulata* (SNr) and of hα-syn + fibres in the striatum, were performed using the ImageJ software (version 1.8.0, NIH). For the TH analysis, 5–7 striatal and 2–4 nigral sections per animal were used and the measured values were normalised for nonspecific background staining by subtracting values obtained from the corpus callosum or the basal part of the cerebral peduncle. The data are presented as a percentage of the mean of both hemispheres of the empty vector group. For hα-syn densitometry analysis, 5–8 striatal slices were used and the measured values normalised by subtracting values from the corpus callosum. The data were correlated with the balance beam outcomes.

### Statistical analysis

GraphPad Prism (v. 8; GraphPad Software, Inc.) was used for statistical analysis. The level of statistical significance was set at p < 0.05 and multiple t-tests were carried out using the Holm–Sidak method. Data are presented as group means ± standard error of the mean (SEM) of n mice. Striatal viral hα-syn overexpression and balance beam outcomes were correlated using the Pearson correlation coefficient and setting the statistical significance at p < 0.05.

## Results

### Manual analysis of motor performances of A53T hα-syn mice in the balance beam test

**Figure 2:**
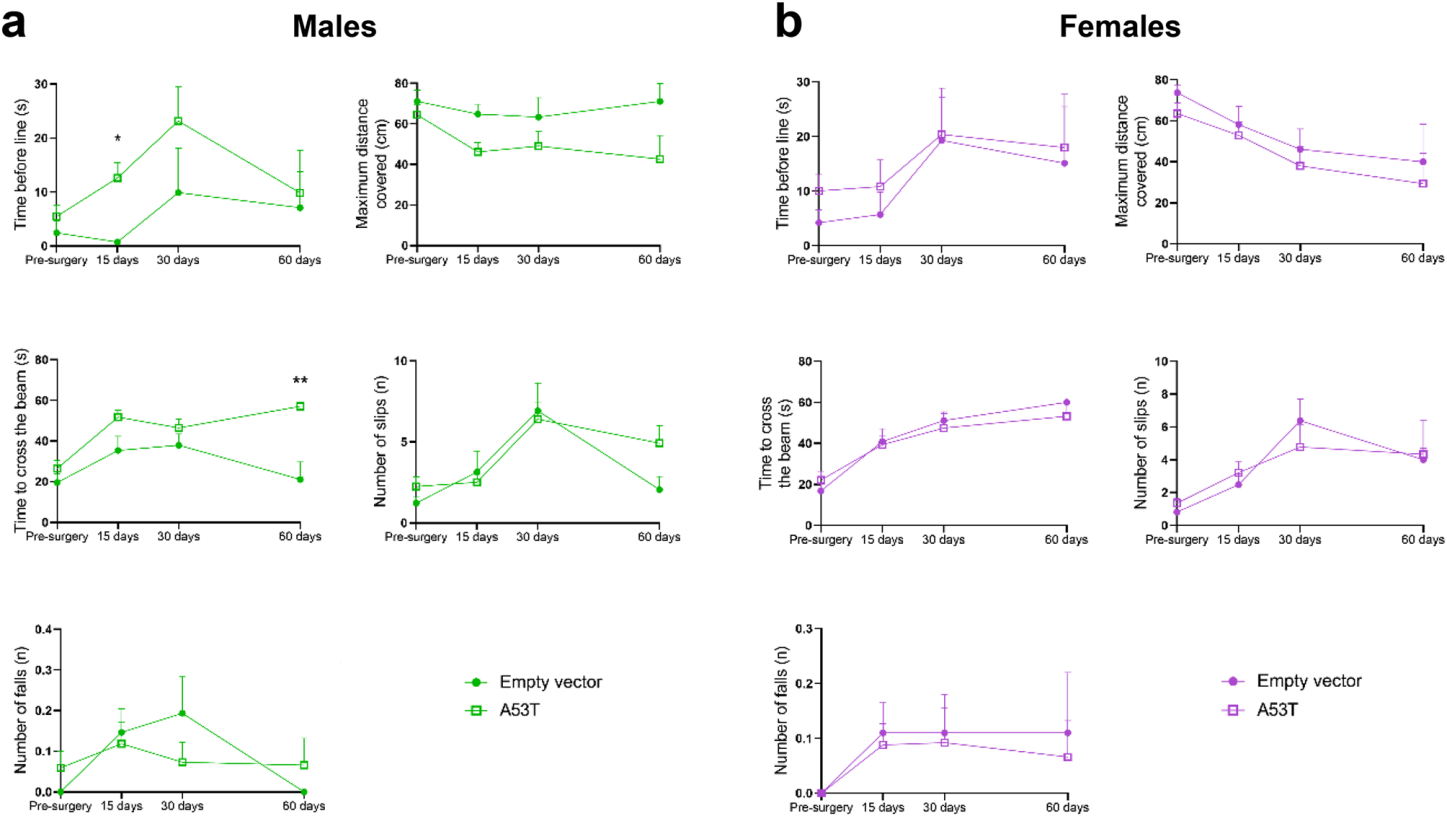
Manual analysis of the balance beam test. Time-course study of the performances of male (**a**) and female (**b**) A53T mutated hα-syn mice on the balance beam test, assessing the time spent before the start line (s), the maximum distance covered on the beam (cm), the time to cross the beam (s), the number of slips (n) and the number of falls (n). Data are expressed as mean ± SEM. *p < 0.05, **p < 0.01 (multiple unpaired t-tests, Holm–Sidak method).

We conducted a time-course study to assess changes in A53T hα-syn mouse motor performances in the balance beam test. The time spent before the start line, the time needed to traverse the beam, the maximum distance covered and the number of slips and falls were manually quantified from videos. At 15 days post-surgery, male A53T hα-syn mice spent more time before the start line than the male empty vector mice (12.62 ± 2.79 s vs. 0.74 ± 0.37 s; p < 0.05; Fig. 2a) and at 60 days post-surgery, they needed significantly more time to cross the beam than empty vector animals (57.13 ± 2.08 s vs. 21.11 ± 8.62 s; p < 0.01; Fig. 2a). No statistically significant differences were found in these manually analysed parameters between females injected with A53T hα-syn or with the empty vector (Fig. 2b).

### Automated procedure to detect walking behaviour in the balance beam test

#### Body-part tracking using DeepLabCut

**Figure 3:**
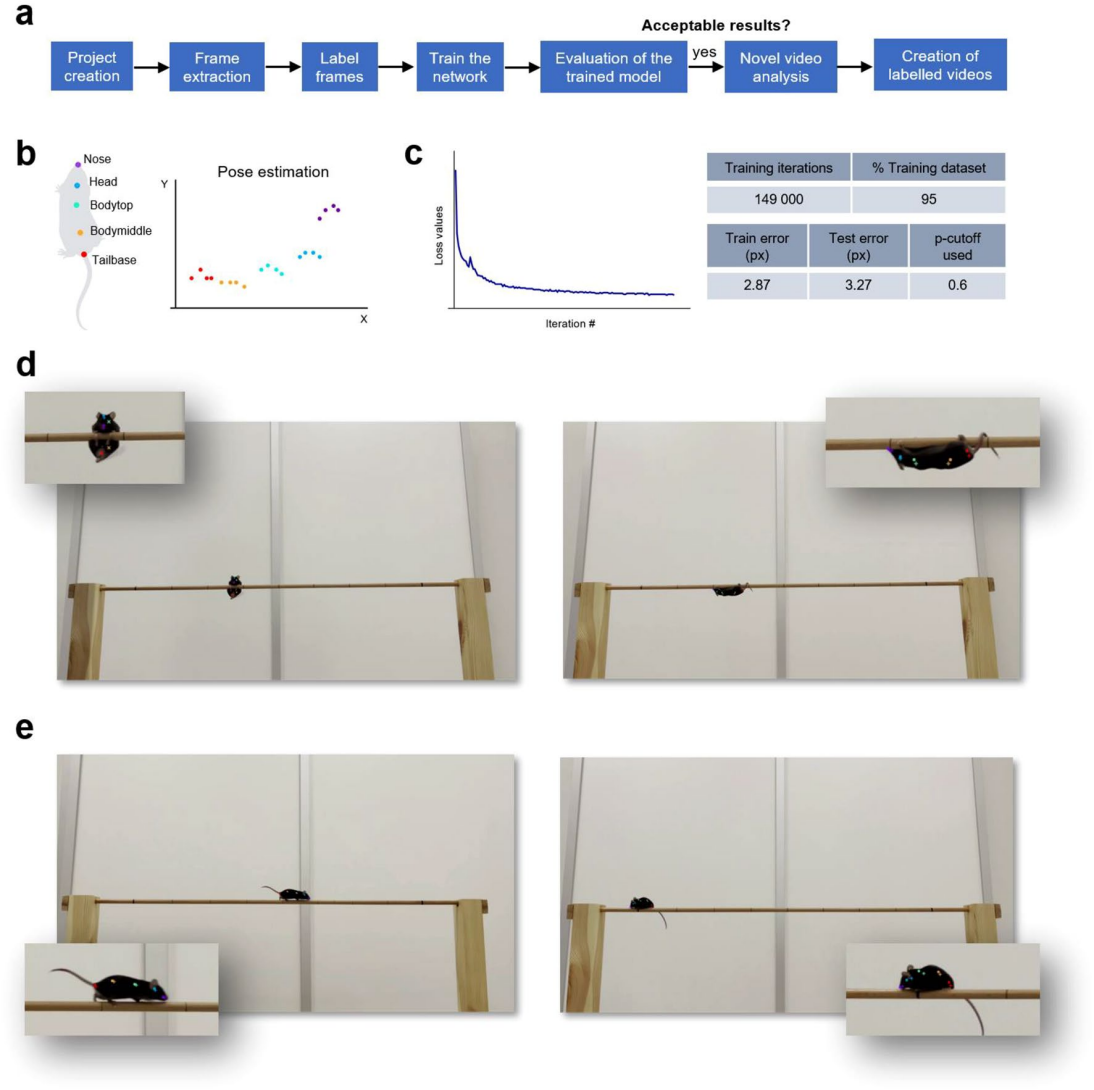
Automated body-part tracking using DeepLabCut. (**a**) DeepLabCut pipeline. (**b**) Schematic overview of the 5 mouse body-parts annotated for pose estimation (colour-coded keypoints) (left). x versus y coordinates of the body-parts (right). (**c**) Training and performance of the network. The network was trained for 149,000 iterations, until the loss values plateaued (left) and the model was evaluated to obtain train and test errors in pixels (right). (**d**,**e**) Examples of labelled frames (training (**d**) and test (**e**) images) created to verify whether DeepLabCut labels matched human-level accuracy. Human annotations are plotted as a “+” symbol and DeepLabCut predictions either as a dot for confident predictions (likelihood > p-cutoff), or as a “x” (likelihood ≤ p-cutoff).

Videos acquired from Camera 2 were first analysed using DeepLabCut for markerless pose estimation (Fig. 3a). Frames were extracted using k-means clustering and then manually labelled for the following 5 body-parts: nose, Head, Bodytop, Bodymiddle and Tailbase (operational definitions are shown in Supplementary Table 1). Body-parts of interest were annotated with high stringency, in order to constitute labelled data CSV files, containing the x, y coordinates of each keypoint (matrix) (Fig. 3b). These files were combined and split into training and test datasets following a 95:5 train/test ratio. The network was trained with the train data, until the loss function plateaued at 149,000 iterations (Fig. 3c). The model was trained to learn how to find the points and the associated loss function stabilising indicated that the model stopped learning and could not optimise more (loss values getting lower, showing that the model is matching the data well). Next, the test data was used to evaluate the performance of the trained network to check the quality of the model. A train error of 2.87 pixels and a test error of 3.27 pixels were achieved with a cutoff value of p = 0.6. Labelled images (both training and test images) were created to visually check how closely the DeepLabCut labels matched the labels placed by the human annotator (Fig. 3d,e). Next, novel videos were analysed to measure how well the model generalises on new data (whether the DeepLabCut predictions for labelling frames are correct on unseen data). The trained network snapshot weights were used for the analysis and trajectories were plotted comparing all body-parts, each identified by a unique colour scheme, retained in all plots. The optional steps of outlier extraction and refining were not run as the model was found to generalise well on new videos (representative video of novel video analysis in DeepLabCut is shown in Supplementary Fig. 1a–d).

#### Behavioural classification using SimBA (analysis of walking)

**Figure 4:**
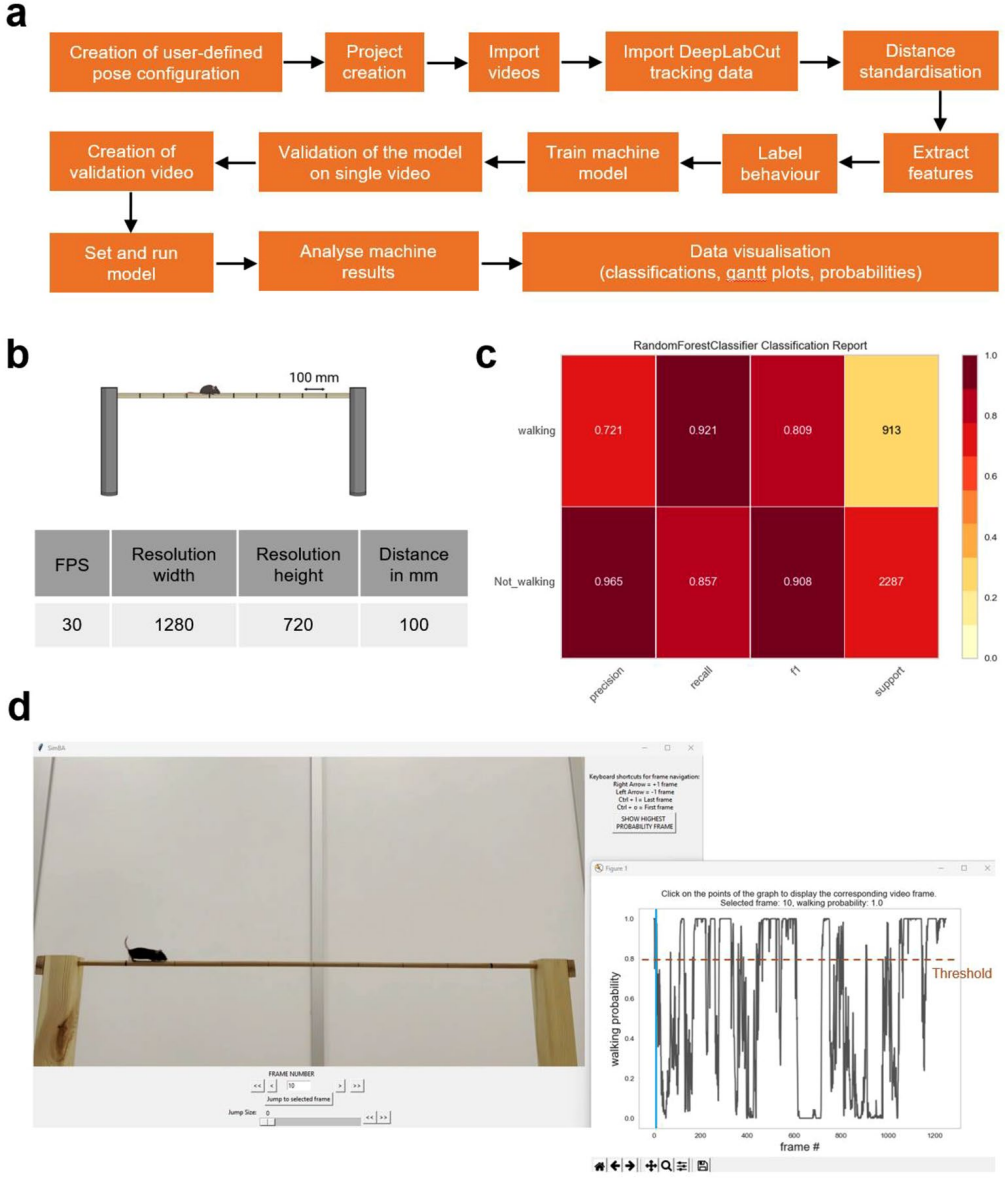
Automated behavioural classification using SimBA. (**a**) SimBA pipeline. (**b**) Configuration of video parameters and distance standardisation. The length between two hand-marked lines along the beam was used to define a standardised distance of 100 mm. (**c**) Classification report truth table generated in SimBA for the analysis of walking, displaying the precision, recall, F1 and support numerical scores and an integrated colour-coded heatmap. (**d**) Optional validation step to determine the optimal discrimination threshold and minimum behaviour bout length in the model detecting walking behaviour. Frame window displaying the validation video and controls to navigate through the frames to verify whether the selected frame contains or not the classifier behaviour (left). Graph window with an interactive graph showing model prediction walking probability versus frame numbers (right). Blue line shows the highest probability frame (selected frame: 10; walking probability: 1.0). The discrimination threshold was set to 0.8 and minimum bout length to 100 ms (corresponding to 3 consecutive frames containing the classifier behaviour).

Pose estimation output data files from DeepLabCut, saved in CSV format, were imported into SimBA for behavioural annotation, classification and analysis (Fig. 4a). A project configuration (config) file was generated with 1 predictive classifier (“walking”), using a new user-defined pose config file, matching the DeepLabCut body-part labels (classifier operational definition detailed in Supplementary Table 2). Video meta parameters were set (30 frames per second (fps); resolution 1280 × 720) and euclidean pixel distances were standardised to millimetre distances by entering a known distance (in mm) in a video frame and calculating the distance in pixels per mm. Here, the length between two hand-marked lines along the beam was used to define a standardised distance of 100 mm (Fig. 4b). Features were extracted based on body-part coordinates in each frame, frame rate and pixel measurement values, to obtain a larger set of features used for behavioural classification. The classifier was built by annotating videos in the SimBA event logger for the absence or presence of the walking behaviour. Limb movements and forward body translation components were used to distinguish walking behaviour from other behaviours not resulting in a displacement of the animal body along the beam (e.g. turning, sniffing, grooming, pause).

Next, a single machine model was trained with a random forest algorithm and a 80:20 ratio was used to split the annotated frames into a train:test dataset (see Supplementary Fig. 2). Random undersampling was selected as a class re-balancing method as the predicted behaviour was found to be sparse (e.g. present in 1–10% of the total number of frames in the videos used for behavioural annotation). Frames containing the behaviour of interest (presence of walking) were imbalanced compared to frames absent of that behaviour, following a 2:1 class weight ratio for present:absent behaviours. The model was subsequently evaluated to assess its performance and a classification report was created to provide a deeper intuition of the classifier behaviour over global accuracy (Fig. 4c). Classification metrics were obtained for the presence and absence of the classifier (“walking” and “not_walking”, respectively). The precision of the model, measuring the classifier’s exactness, was found to be higher for the “not_walking” class in comparison to the “walking” class (0.965 vs. 0.721, respectively), while the recall metric, denoting the classifier’s completeness, was slightly higher for the “walking” class than the “not_walking” class (0.921 vs. 0.857, respectively). The weighted harmonic mean of the precision and recall scores is shown in the F1-score, measuring the overall model performance from 0 to 1, where the best score is 1. We observed high F1-scores for both classes (0.809 for “walking” and 0.908 for “not_walking”), denoting a good-quality classifier. Support values for the model, reflecting the number of actual occurrences of the class, were mildly imbalanced with a proportion of the minority class (“walking”) of 29% (Fig. 4c).

The SimBA model was next evaluated on new (out-of-sample) data to determine the optimal discrimination threshold, i.e. the level of probability required to define that the frame belongs to the target class, as well as the minimum duration of a walking event to set the model. By navigating through the frames in the graph and frame windows in SimBA, we set a discrimination threshold to 0.8 so that frames with a probability of containing the behaviour of 0.8 or above are classified as containing the behaviour walking (Fig. 4d). The minimum behaviour bout length was set to 100 ms (corresponding to 3 consecutive frames with the behaviour present). The model was then run and machine predictions were analysed in SimBA, generating gantt and probability plots for all the videos in the project (an example of SimBA output data for a representative video is provided in Supplementary Fig. 1e,f).

### Automated analysis of progressive motor deficits in the balance beam test in A53T hα-syn mice

**Figure 5:**
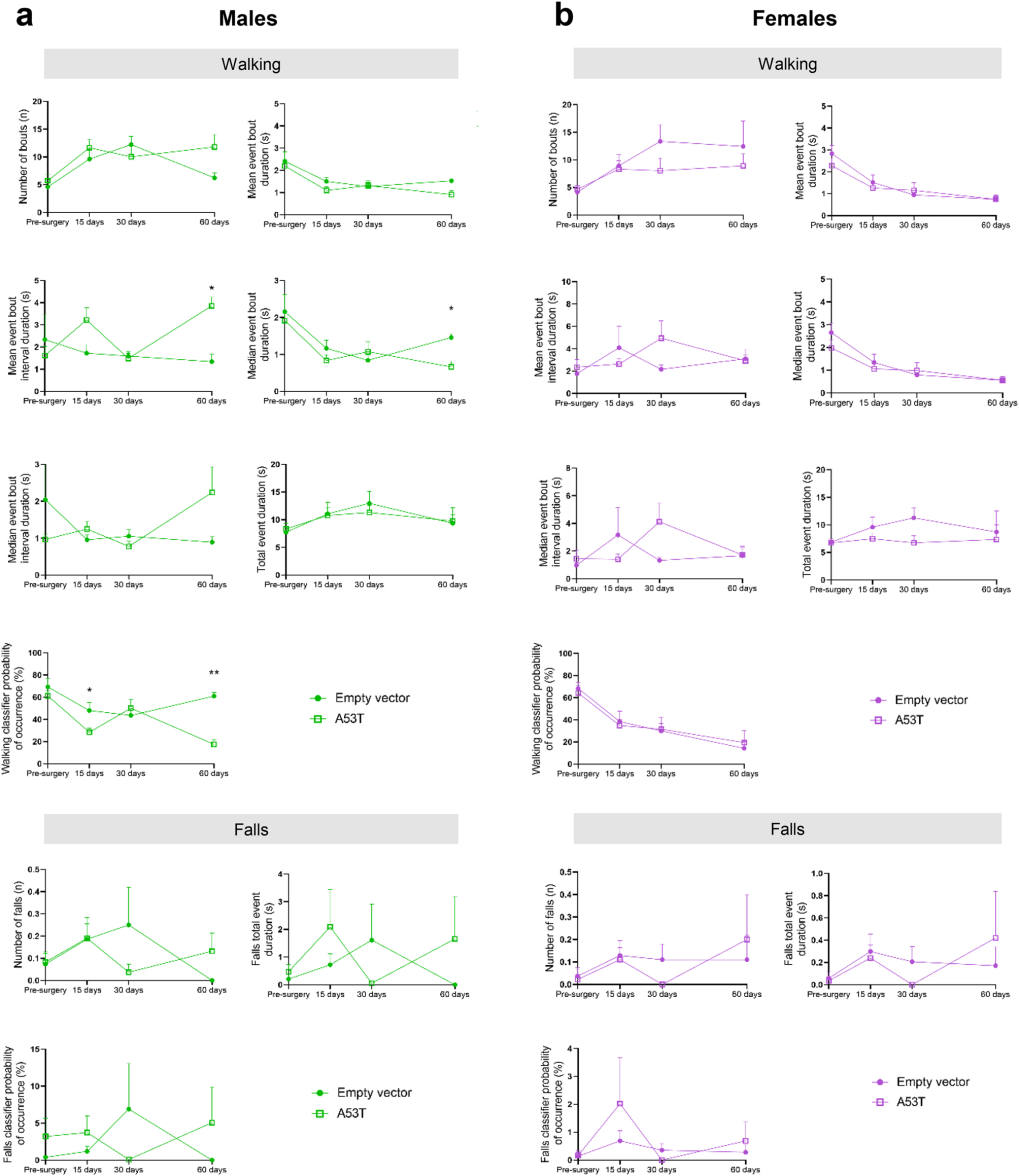
Automated analysis of the balance beam test. Temporal changes in the walking analysis in the number of behavioural bouts (n), mean event bout duration (s), mean event bout interval duration (s), median event bout duration (s), median event bout interval duration (s), total event duration (s), walking classifier probability of occurrence (%), and in the falls analysis in the number of falls (n), falls total event duration (s) and falls classifier probability of occurrence (%) in male (**a**) and female (**b**) A53T mutated hα-syn mice. Data are expressed as mean ± SEM. *p < 0.05, **p < 0.01 (multiple unpaired t-tests, Holm–Sidak method).

Using the combination of DeepLabCut and SimBA for pose estimation and supervised behavioural classification respectively, we obtained an in-depth automated analysis of videos recorded on Camera 2. We assessed the number of behavioural bouts (i.e. uninterrupted walking episodes), the mean event bout duration, mean event bout interval duration (corresponding to the animal not walking, i.e. frozen, or stopped walking), median event bout duration, median event bout interval duration, total event duration (i.e. the time the mouse spent walking), the walking classifier probability of occurrence (percentage of walking), number of falls, and the total event duration and the classifier probability of occurrence of falls (operational definition of falls detailed in Supplementary Table 2). At 60 days post-surgery in male A53T hα-syn mice, the mean event bout interval duration was significantly higher and the median event bout duration significantly lower than in male empty vector animals (3.86 ± 0.39 s vs. 1.34 ± 0.33 s; 0.66 ± 0.15 s vs. 1.46 ± 0.11 s, respectively; p < 0.05; Fig. 5a). The walking classifier probability of occurrence decreased significantly in male A53T hα-syn mice at 15 and 60 days post-surgery in comparison to empty vector mice (28.42 ± 3.74% vs*.* 48.07 ± 7.01%, p < 0.05; 17.52 ± 4.32% vs*.* 61.01 ± 3.37%, p < 0.01, respectively; Fig. 5a). No statistically significant differences were found in the automated analysis between A53T hα-syn and empty vector female mice (Fig. 5b).

### Comparison of manual and automated analyses

**Figure 6:**
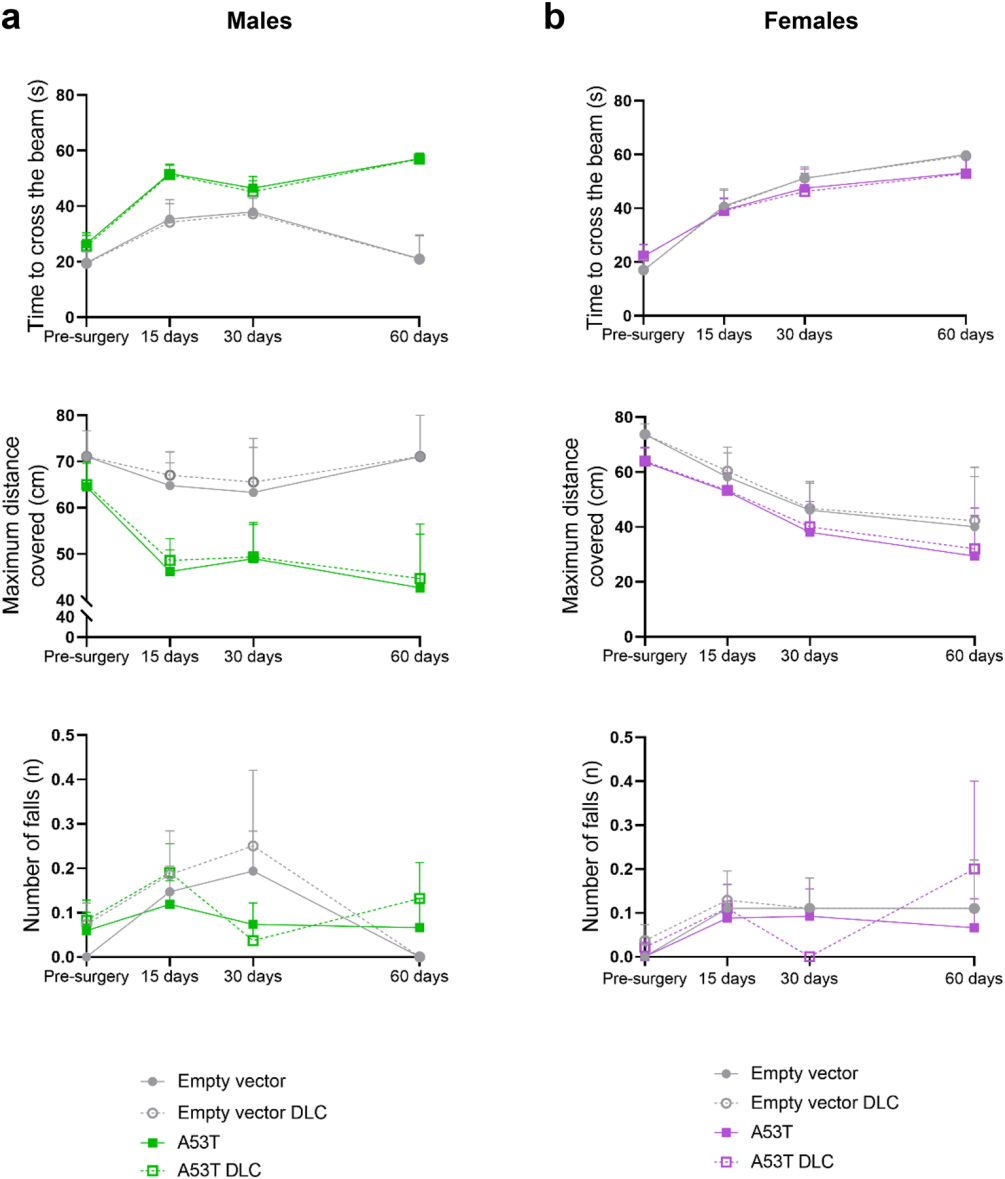
Comparison between manual and automated analyses. Comparison between manual scoring and the automated procedure for the time to cross the beam (s), the maximum distance covered (cm) and the number of falls (n) in male (**a**) and female (**b**) A53T mutated hα-syn mice. Data are shown as mean ± SEM.

In SimBA, ROIs were used to fractionate the different sections of the beam in order to measure the time to cross the beam (s) and the maximum distance covered (cm) in an automated manner and compare them with the manual approach. Falls were also manually quantified and a model in SimBA was created for automated analysis of the number of falls, for comparison between both manual and automated approaches. No statistically significant differences were found between manual and automated analyses in these parameters, in neither males nor females (Fig. 6).

### Histological verifications

#### Transgene expression following AAV-vector injection

To verify the expression of A53T mutated hα-syn at 15, 30 and 60 days post-surgery, immunohistochemistry for α-syn was performed in striatal and nigral slices. Overexpression of A53T hα-syn led to increasing immunoreactivity over time, in both the striatum and SN (representative images shown in Supplementary Fig. 6a), and no hα-syn expression was observed in empty vector injected animals.

#### Correlation between striatal hα-syn overexpression and balance beam outcomes

Striatal hα-syn optical density was correlated with the balance beam outcomes in order to determine whether mice with more viral overexpression showed more motor alterations. Positive correlations were observed in males in the time needed to cross the beam and the mean event bout interval duration, and a negative correlation in the walking classifier probability of occurrence (Supplementary Fig. 7). Nevertheless, no significant correlations were found between striatal hα-syn optical density and the balance beam outcomes in females (data not shown).

#### Maintenance of nigrostriatal dopaminergic innervation

To evaluate nigrostriatal degeneration, immunohistochemistry for TH was performed and the mean optical density of both hemispheres of A53T hα-syn mice was analysed in comparison to empty vector mice. In the analysis of dopaminergic projections to the striatum, no differences were found at 15, 30 and 60 days post-surgery in neither males nor females (Supplementary Fig. 6b). We also assessed the dendritic arborisation of the SNr derived from DA neurons of the SNc. Surprisingly, we observed that at 30 days post-surgery, nigral TH + fibres in male A53T hα-syn mice were 20% higher than in male empty vector mice. This may be due to subtle mechanical damages caused by the injection that were observed in some male empty vector mice. In females, there were no significant differences in nigral TH + fibres, as assessed by optical densitometry (Supplementary Fig. 6c).

## Discussion

Early detection of motor impairments in animal models of PD is becoming essential for the preclinical development of new therapeutic agents capable of modifying the course of the disease. Currently, PD remains incurable, with late clinical diagnosis based on motor symptomatology preventing the use of neuroprotective strategies. Accurate behavioural analysis of subtle motor deficits in PD mice has remained a challenge due to limitations related with conventional manual quantification methods, persisting in being extremely time-intensive, prone to human error and presenting low replicability within the scientific community.

The overall goal of this study was to combine manual and automated analysis approaches to quantify walking behaviour in video recordings of mouse performances in the balance beam test. We developed a model for pose estimation to accurately track mouse body-parts using the video analysis software, DeepLabCut. We then imported the tracking output data into SimBA for behavioural annotation and automated behavioural classification and analysis. We obtained the duration and frequencies of behavioural bouts (uninterrupted, continuous walking episodes lasting 100 ms or longer), which would have been difficult to achieve by a manual scorer. Indeed, our automated analysis revealed the pattern of mouse walking behaviour along the beam and its percentage of occurrence in each trial, relating to disrupted patterns when the continuity of the walking flow was interrupted when the animal was pausing/stopping. This is of interest in a model of PD as gait and balance disturbances are common features of PD patients, with heterogeneous mobility impairments that remain difficult to predict^19^. Investigating and developing prognostic models to detect gait and balance disturbances in PD is therefore of great importance. With our automated model, we showed that subtle behavioural changes induced by the overexpression of A53T mutated hα-syn were detectable before nigrostriatal dopaminergic degeneration was observed. These motor changes could be due to dysfunction in dopaminergic transmission in PD occurring before Lewy body pathology and neurodegeneration, as previously reported^20,21^. Motor impairments observed in animals injected with the empty viral vector were hypothesised to maybe be due to mechanical damage caused by the injection.

Current research is primarily investigating the earliest stages of α-syn-induced pathological synaptic changes to better understand the disease mechanisms^22^. In a recent study^23^, no dopaminergic degeneration was observed after 12 weeks of unilateral nigral injection using C57BL/6 mice and AAV1/2-A53T-α-syn virus. Authors of that study justified that the overexpression of α-syn can induce other changes that lead to the emergence of motor symptoms, such as neuroinflammation or altered synaptic activity that prevent normal neuronal function. Our PD mouse model may therefore be considered at an early phase, without a clear manifestation of neurodegeneration at the level of the TH + fibres. We were able to detect subtle motor alterations that were correlated with the overexpression of α-syn, even though no neurodegeneration of TH + fibres was detected. Our results are thus promising, as we are detecting motor changes in PD mice before neurodegeneration even occurs, which is paramount for future testing of neuroprotective agents capable of changing the course of the disease.

Interestingly, we observed significant time-course differences in A53T hα-syn male performances for the time to traverse the beam in the manual analysis. Previous studies have demonstrated changes in the time needed to cross the beam, but neither the PD animal model used, nor the times at which the behavioural test was performed, nor the protocol for performing the balance beam test were the same^24–26^. Other studies based on α-syn used transgenic mice overexpressing human wild-type α-syn and reported time-course changes in the time to traverse the beam^27,28^ and in the errors per steps^27^. To our knowledge, this is the first article where the balance beam test is performed in a PD model based on the overexpression of α-syn induced by adeno-associated viral vectors.

Additionally to analysing the pattern of the walking behaviour in a mouse model of early PD, we compared manual and automated analyses for the time to cross the beam, the maximum distance covered and the number of falls. We found no significant differences between manual and automated analyses for each parameter, thus providing a promising insight for future studies using the balance beam test, where the manual input could be reduced and replaced by our automated procedure.

Previous studies have used DeepLabCut for gait analysis in the balance beam test in mice, piglets and preterm pigs^29–31^. However, as far as we know, SimBA has not yet been used for behavioural classification of animal performances in the balance beam test. We therefore provide a novel approach to analysing animal performances in this motor task, by presenting an automated procedure using DeepLabCut and SimBA. Whereas a previous study utilised both of these softwares to analyse the open field test in 6-hydroxydopamine animal models of PD^32^, to the best of our knowledge, automated behavioural analysis using DeepLabCut combined with SimBA has not yet been achieved in a mouse model of PD in the balance beam test.

Although our DeepLabCut and SimBA models were developed to track C57BL/6J mouse performances in the balance beam test (camera placed laterally to the beam), the neural network could be expanded to generalise well on other rodent models that may differ in coat-colour or size, or on video recordings of lower quality^18^. A limitation of our procedure was that videos were analysed post-behavioural sessions, requiring storage and pre-processing, which was found to be time-intensive. This could be addressed by using a real-time pose estimation software package, such as DeepLabCut-Live!^33^.

Additionally, our study could be improved by adding an additional camera to our experimental set-up, in order to view the mouse on the beam from the front as well as our current rear camera angle. Capturing both hind paw movements simultaneously with different camera perspectives could enable us to incorporate the quantification of hind paw slips into our machine models, with the aim to reduce manual scoring. Indeed, in our study, we were unable to automate hind paw slip quantification using videos from our current camera angles. We were able to add keypoints onto the mouse hind paw in DeepLabCut (data not shown) but the model created in SimBA could not accurately assess the slip counts due to hind paws not always being visible in the video frame, and due to the variability and complexity of a slip itself. Another study^29^ presented an automated way for slip quantification in a mouse study on the balance beam test by studying hind paw trajectories and rhythmicity of gait pattern. However, this study was limited to one camera angle lateral to the beam, preventing the analysis of both hind paws. Nevertheless, with additional camera angles, we could expand our current model for a more in-depth pose estimation analysis than presented in this study. Furthermore, we could pursue the movement analysis of PD mice by adapting our automated model to include additional behavioural classifiers in SimBA (e.g. turning, freezing) to study the duration and frequencies of these behaviours in the videos. Behavioural studies could also be carried out at a later stage, thus increasing the time post-injection, to potentially assess motor impairments and obtain a level of neurodegeneration, as the experimental times used in this study may not be sufficient to observe neurodegeneration.

### Supplementary Information

Supplementary Information.

## Data Availability

The datasets generated during and/or analysed during the current study are available from the corresponding author on reasonable request.
