# Supplementary material for: Automated procedure to detect subtle motor alterations in the balance beam test in a mouse model of early Parkinson’s disease
Source: Sci Rep. 2024 Jan 9;14:862. doi: 10.1038/s41598-024-51225-1 (PMC10776624; doi:10.1038/s41598-024-51225-1)
Supplement: Supplementary file 1 — Supplementary Information. [file 41598_2024_51225_MOESM1_ESM.docx]

SUPPLEMENTARY INFORMATION

**Automated procedure to detect subtle motor alterations in the balance beam test in a mouse model of early Parkinson’s disease**

Raphaëlle Bidgood^1#^, Maider Zubelzu^1, 2#^, Jose Angel Ruiz-Ortega^1, 2^ and Teresa Morera-Herreras^1, 2*^

^1^Department of Pharmacology, University of the Basque Country (UPV/EHU), Leioa, Bizkaia, Spain.

^2^Autonomic and Movement Disorders Unit, Neurodegenerative diseases, Biobizkaia, Barakaldo, Bizkaia, Spain.

#: These authors contributed equally to this work

**CONTENTS:**

**Supplementary Tables (2)**

**Supplementary Figures (7)**

* Corresponding author:

**Teresa Morera-Herreras**

Department of Pharmacology, Faculty of Medicine and Nursing, University of the Basque Country (UPV/EHU)

E-mail address: [teresa.morera@ehu.eus](mailto:teresa.morera@ehu.eus)

Postal address: Department of Pharmacology, Faculty of Medicine and Nursing, University of the Basque Country (UPV/EHU), Barrio Sarriena s/n, 48940-Leioa, Spain

Telephone number: +34 94 601 58 91

ORCID number: 0000-0002-7601-4914

**Supplementary Table 1. Operational definitions of mouse body-parts during labelling with DeepLabCut.**


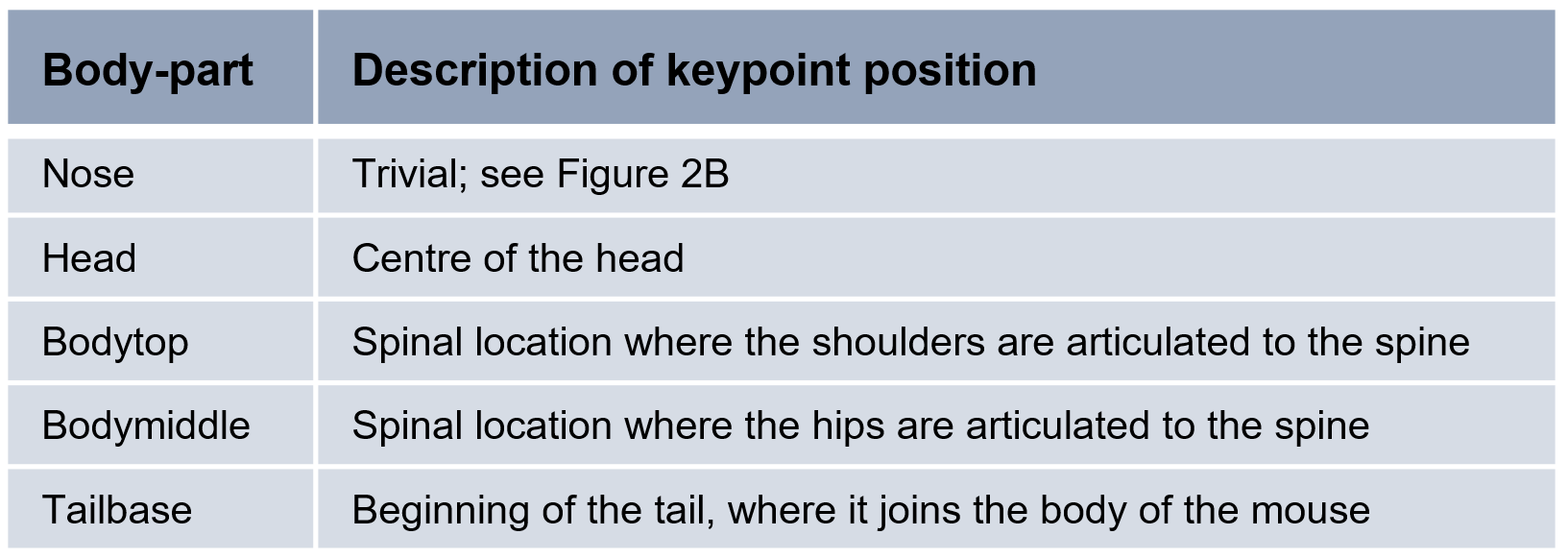


**
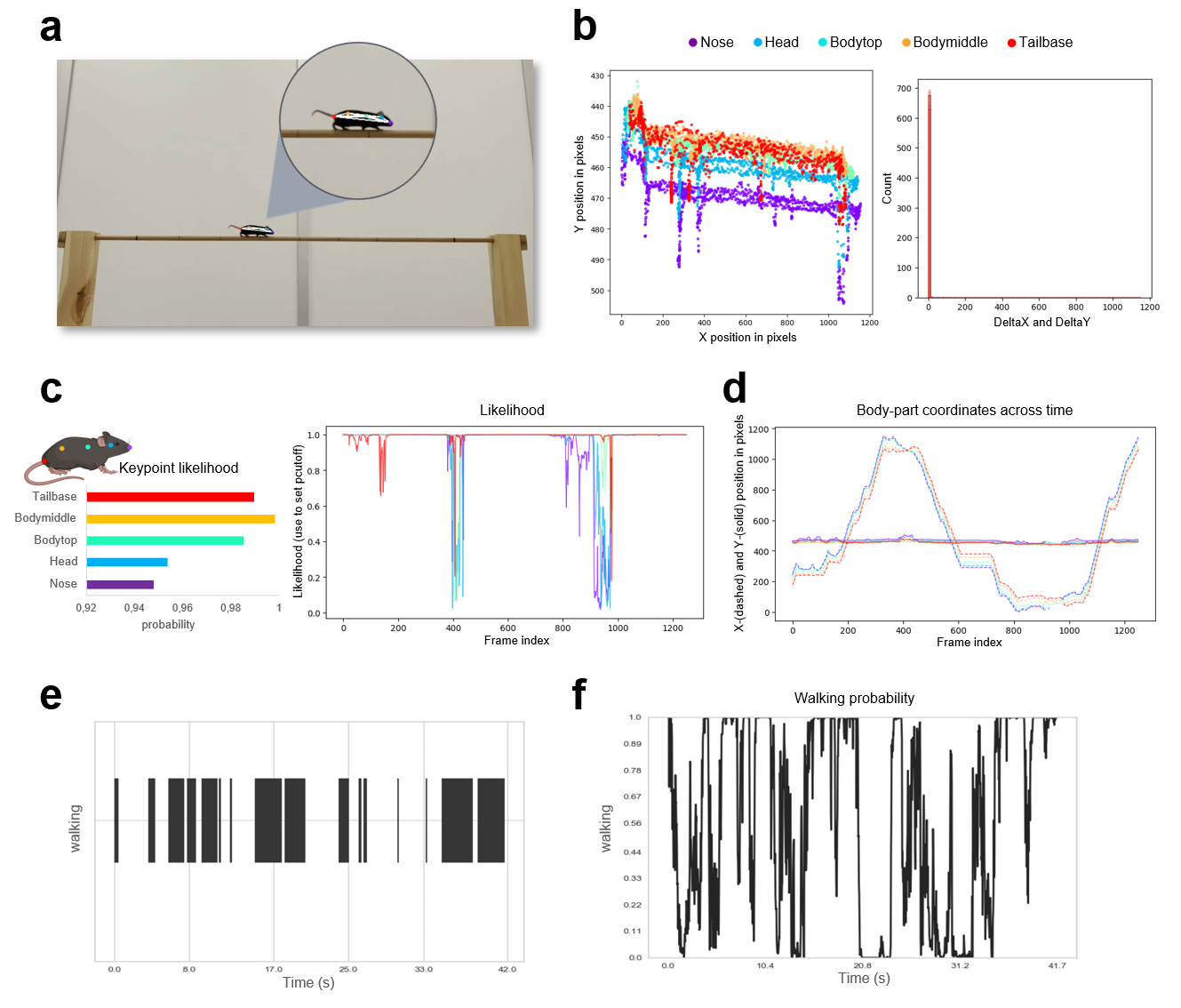
**

**Supplementary Figure 1. Representative video of automated analyses of mouse walking behaviour in the balance beam test, using DeepLabCut and SimBA. (a)** Labelled video created using DeepLabCut, showing the 5 keypoints on the animal (Nose, Head, Bodytop, Bodymiddle and Tailbase) as well as a custom skeleton (white) interconnecting the body-parts. The video was taken by Camera 2, placed laterally to the beam. **(b)** Generated plots for the analysed video using DeepLabCut. Trajectory of the mouse, displayed as x versus y coordinates of the body-parts plotted in space over all frames (left). Histogram of consecutive coordinate differences, with values close to zero indicating no jumps in body-part detection across frames (right). **(c)** Keypoint likelihood across all frames of the representative video, shown as means of the body-part probabilities for each video frame (p_Tailbase_: 0.99; p_Bodymiddle_: 0.998; p_Bodytop_: 0.985; p_Head_: 0.954; p_Nose_: 0.948) (left). Body-part likelihoods versus time (ideally, the likelihood stays high), generated using DeepLabCut (right). **(d)** Plot displaying all body-parts across time, with X and Y positions in pixels (dashed and solid lines, respectively) over all frames. Dashed lines reveal the movement of the animal walking along the beam (lateral point of vue from Camera 2), with lower X values corresponding to the mouse located closer to the start line, and higher X values to the mouse closer to the finishing line, marked on the beam. Y values remained unchanged as the animal stayed on the beam. **(e)** Gantt plot created in SimBA displaying the duration and frequency of the walking behaviour in the representative video. **(f)** Probability of occurrence of the behavioural classifier “walking” across frames of the representative video (generated in SimBA).

**Supplementary Table 2. Operational definitions of classifiers for behavioural annotation in SimBA.**

| **Classifier** | **Description** | **Start frame** | **Duration of behaviour** | **End frame** |
| --- | --- | --- | --- | --- |
| 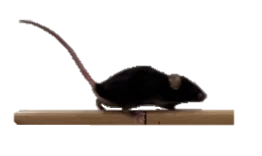  Walking | Limb movements displacing the whole body of the animal to advance along the beam (type of legged ambulation resulting in locomotion). Walking was accompanied by tail movements, aiding the mouse to keep its balance to stay on the beam. Animals were found to vary their gait rhythm and speed depending on their stability (walking and running were not distinguished). | First frame where the body of the mouse is displaced in a forward motion in comparison to the previous frame, accompanied by alternating steps and often, the elongation of the body. | Uninterrupted forward body translation along the beam where a step transitions into another step (continuous limb movement). | First frame where the mouse has stopped alternating steps, resulting in an interruption in locomotion (animal body no longer displaced in a forward manner). |
| 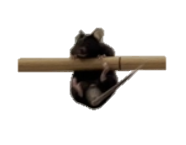  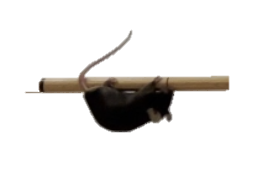Falls | Mouse visibly struggling to stay on the beam and either has both hind paws hanging below the beam, or is clinging onto the beam upside down. | Often starting as a pronounced slip where both hind paws come off the beam and the animal is hanging onto the beam only with its front paws. | Falls duration varied depending on whether the animal was clinging onto the beam (shorter duration) or was upside down (longer duration). | First frame where the animal has managed to get back onto the beam or where it has let go of the beam and fallen. |

**
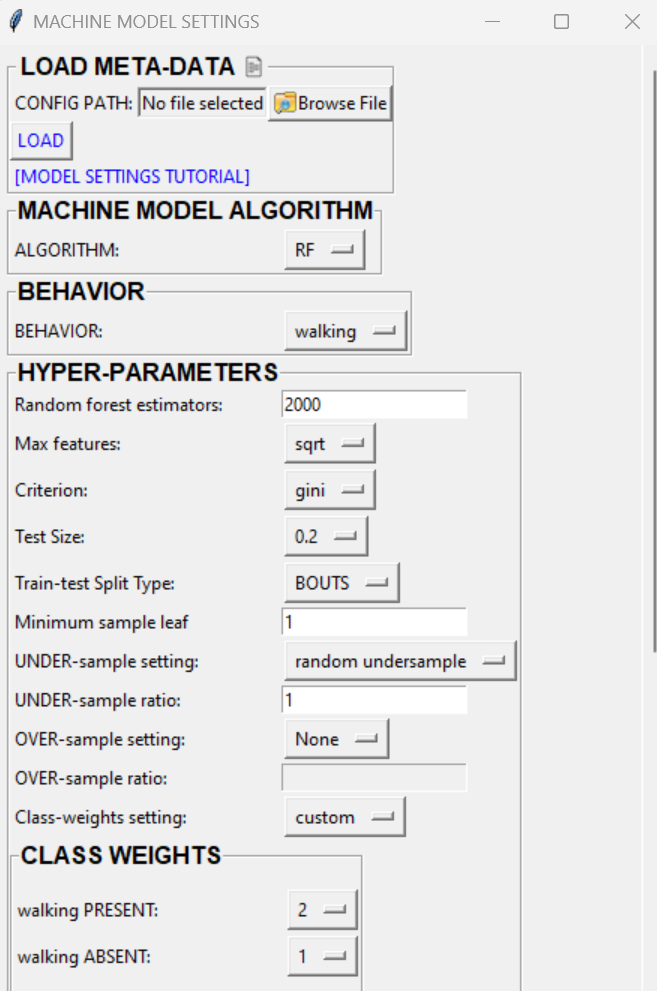

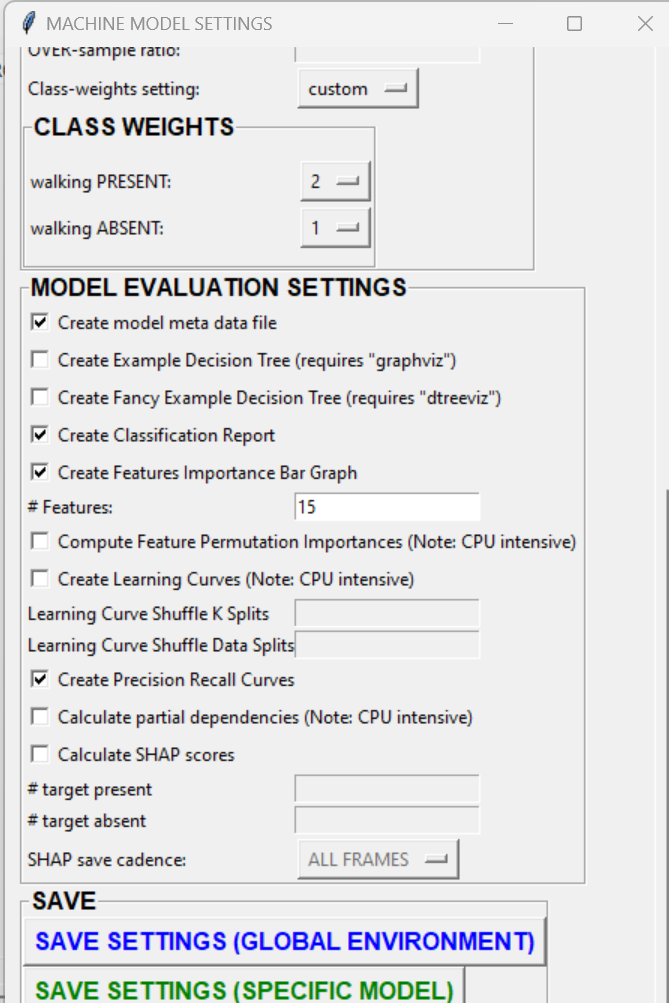
**

**Supplementary Figure 2. Machine model settings in SimBA.**


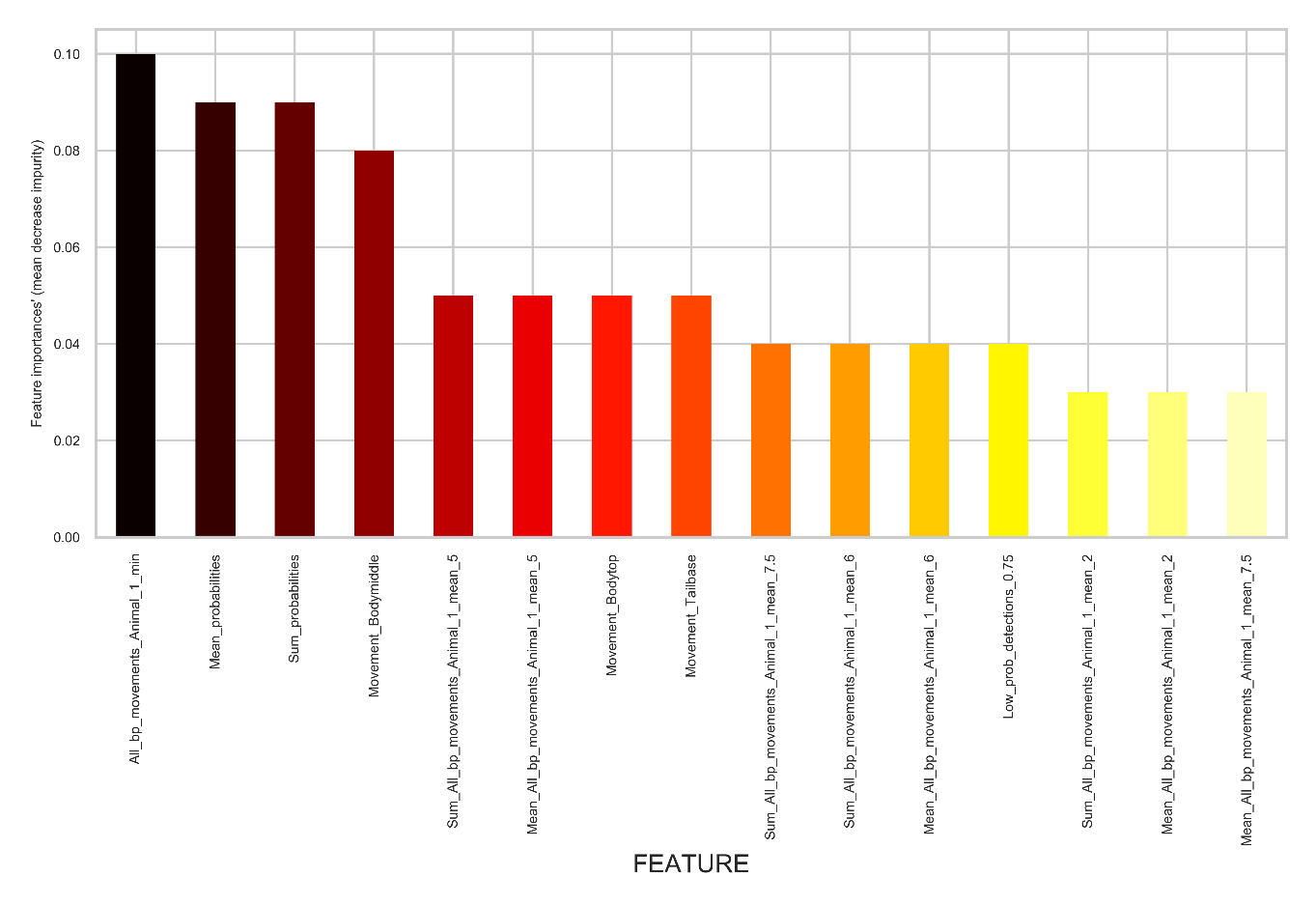


**Supplementary Figure 3. Walking feature importance bar graph.** Bar chart of the top N features based on gini importances, with N=15 (generated in SimBA).

**
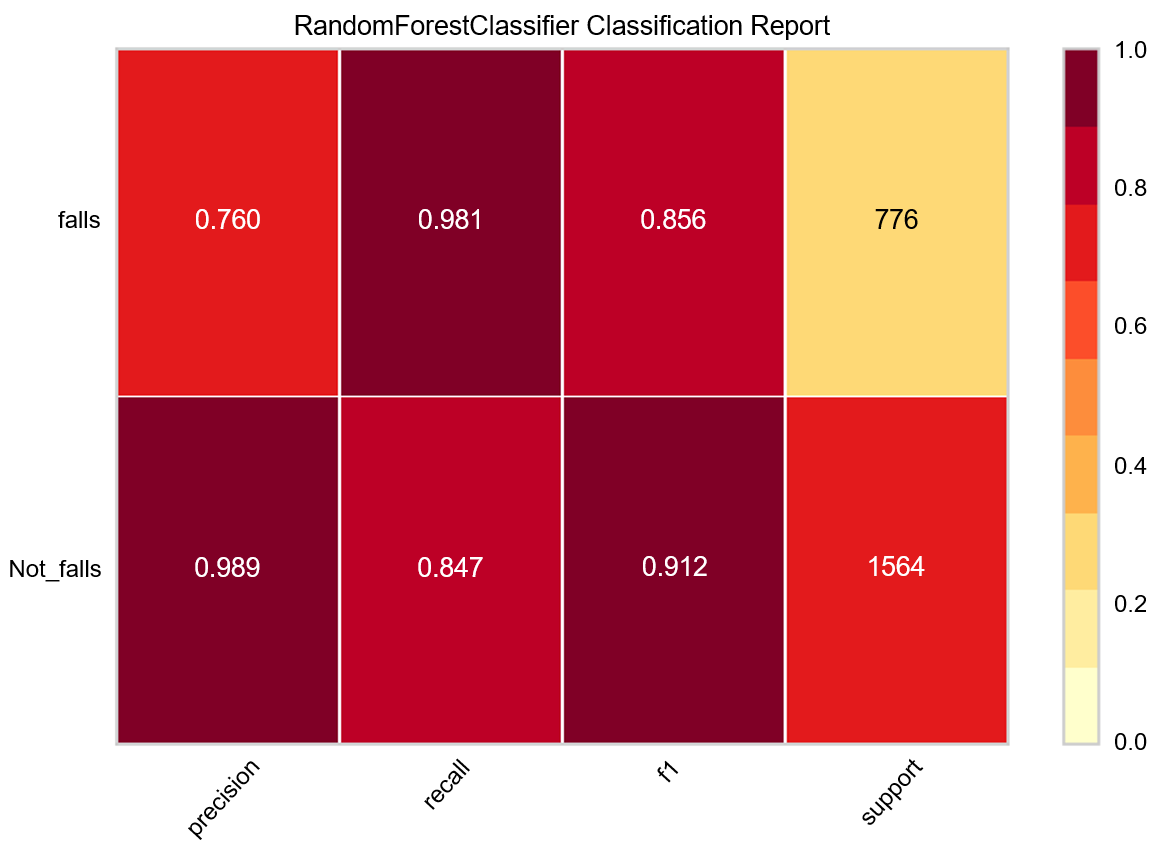
**

**Supplementary Figure 4. Classification report table generated in SimBA (analysis of falls).** Classification report truth table of the automated model detecting falls, displaying performance metric scores (precision, recall, F1 and support numerical scores) and an integrated colour-coded heatmap.

**
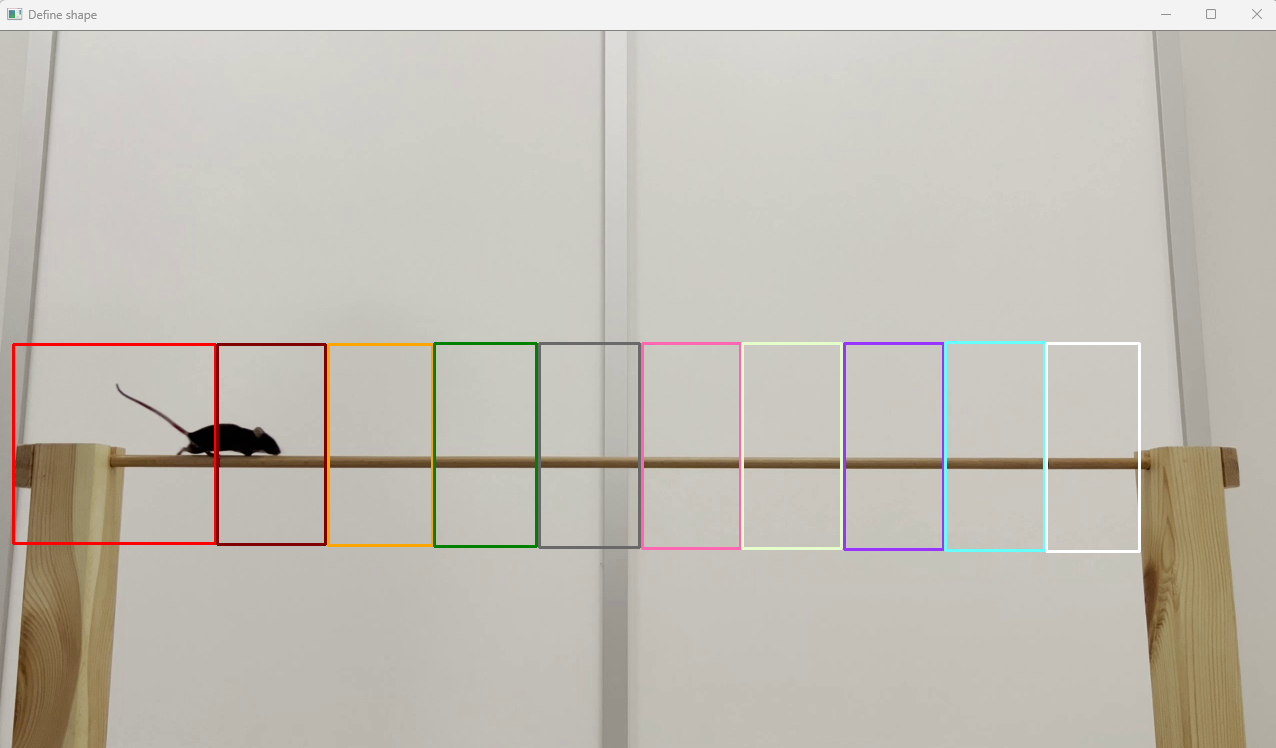
**

**Supplementary Figure 5. Regions of interest (ROIs) in SimBA.** ROIs were drawn in the SimBA ROI GUI to fragment the beam into 10 regions, corresponding to the regions before the start line and after the finishing line and regions on the beam delimited by the hand-drawn lines on the beam (marked every 10 cm).

**
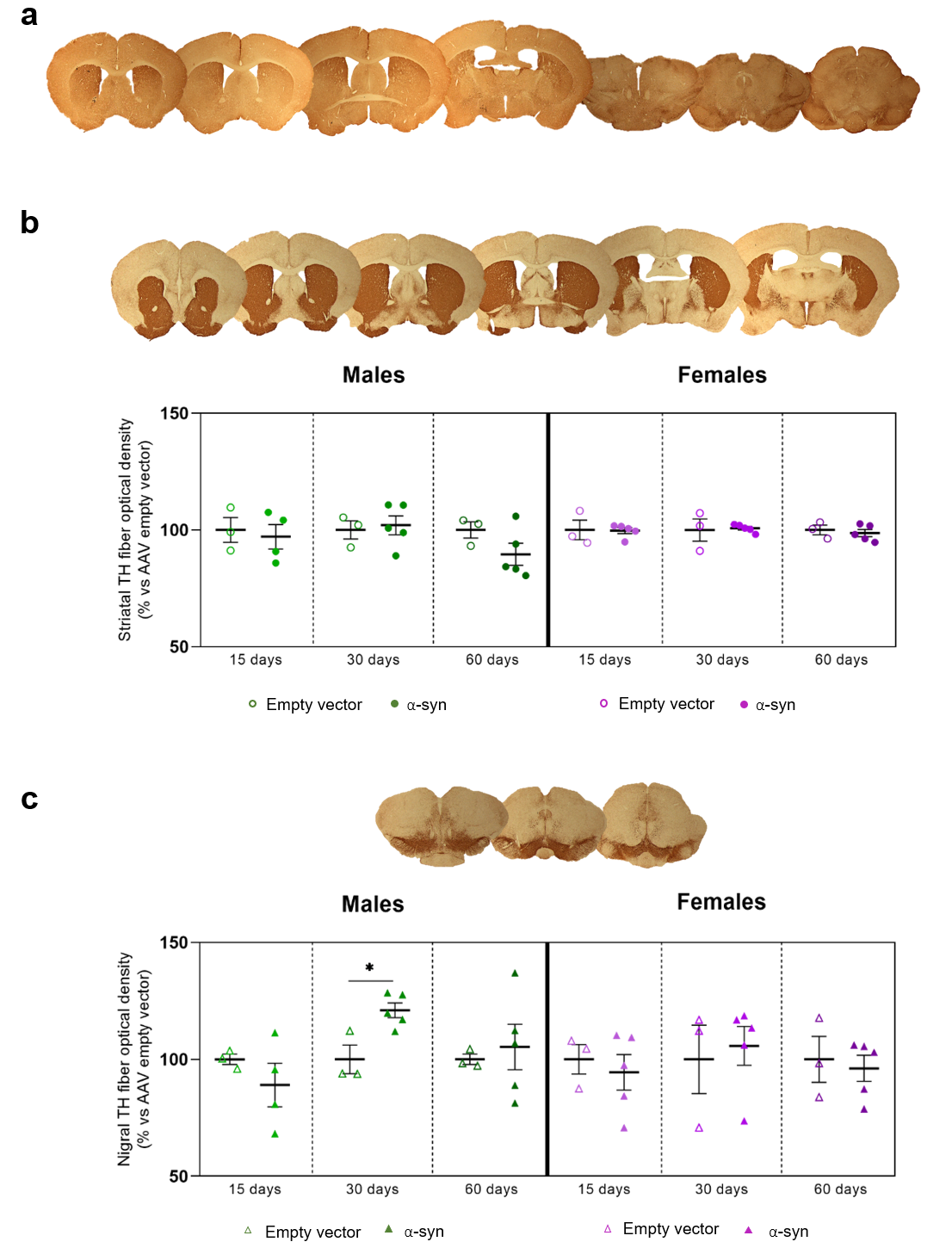
**

**Supplementary Figure 6. Histological verifications. (a)** Representative mouse coronal brain slices showing α-syn immunoreactivity, in both the striatum and SN caused by overexpression of A53T hα-syn. **(b)** Analysis of dopaminergic projections to the striatum. **(c)** Assessment of dendritic arborisation of the SN *pars reticulata* derived from DA neurons of the SNc.


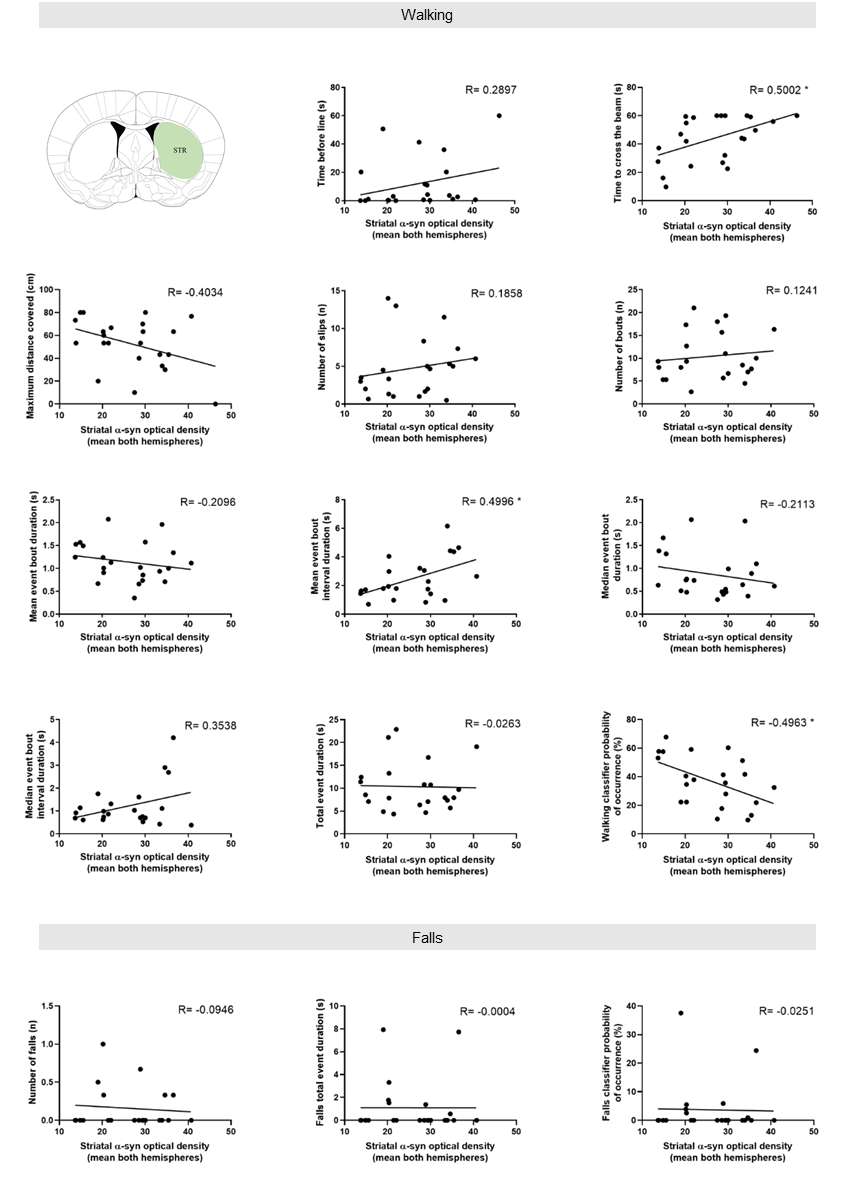
**Supplementary Figure 7. Correlation between striatal α-syn overexpression and balance beam outcomes in male mice.** Striatal α-syn optical density correlated positively with the time to cross the beam and the mean event bout interval duration, and negatively with the walking classifier probability of occurrence in males. *p<0.05
